# Prognostic Value of the C‐PLAN Index in Advanced Esophageal Squamous Cell Carcinoma Patients Treated With Immune Checkpoint Inhibitors

**DOI:** 10.1002/kjm2.70140

**Published:** 2025-12-04

**Authors:** Yi Zhou, Li‐Hua Yang, Jing Tang, Yuan‐Ming Li, Ping Zhao, Zan Li

**Affiliations:** ^1^ Department of Gastroenterology Guangyuan Central Hospital Guangyuan Sichuan China; ^2^ Department of General Surgery Guangyuan Hospital of Traditional Chinese Medicine Guangyuan Sichuan China

**Keywords:** C‐PLAN index, disease progression, esophageal squamous cell carcinoma, immune checkpoint inhibitors, prognosis

## Abstract

This study evaluated the prognostic value of the C‐PLAN index in advanced esophageal squamous cell carcinoma (ESCC) patients receiving immune checkpoint inhibitor (ICI) therapy. A retrospective analysis of 241 eligible patients treated during February 2020 to January 2023 was conducted. Based on the C‐PLAN index, calculated from lactate dehydrogenase (LDH), C‐reactive protein (CRP), performance status (PS), albumin (ALB), and derived neutrophil‐to‐lymphocyte ratio (dNLR), patients were categorized into Good (0–1 points) and Poor (2–5 points) groups. The Poor group exhibited more advanced clinical stages and larger tumor diameters (both *p* < 0.05). The Good group demonstrated a significantly higher objective response rate and disease control rate (both *p* < 0.05), lower progression/death incidence (both *p* < 0.001), and longer progression‐free survival and overall survival (*p* < 0.001). Multifactorial Cox regression analysis revealed that PD‐L1 CPS < 10%, clinical stage IV, and high C‐PLAN score (2–5 points) were independent risk factors for disease progression or death following ICI therapy in patients with advanced ESCC. The C‐PLAN index effectively stratifies prognosis and optimizes therapeutic decision‐making for advanced ESCC. The C‐PLAN index serves as a prognostic factor, providing an objective basis for survival assessment and treatment plan optimization in advanced ESCC patients.

## 1 Introduction

Esophageal cancer (EC) is a highly aggressive malignant tumor of the digestive tract, with squamous cell carcinoma being the predominant histological type [1]. According to GLOBOCAN 2020 data [2], EC ranks seventh in incidence and sixth in mortality among all malignancies, with a generally unfavorable prognosis and a low 5‐year survival rate. Common risk factors linked to the cancer are excessive alcohol consumption and tobacco smoking. Microorganisms, genetic factors, dietary factors, and some other environmental factors may contribute to esophageal squamous cell carcinoma (ESCC) etiopathogenesis [3]. For advanced ESCC, chemotherapy has been the conventional treatment, yet its efficacy remains suboptimal [4]. However, with advancements in medical research, growing evidence suggests promising antitumor effects of immune checkpoint inhibitors (ICIs) [4].

In recent years, ICIs have achieved breakthrough progress in the therapy of advanced ESCC, emerging as a key focus in clinical research [5]. Multiple studies have confirmed the ability of ICIs in substantially improving the survival outcomes of patients with locally advanced or metastatic ESCC [6, 7, 8, 9]. ICIs primarily function by targeting the programmed cell death protein 1/programmed death‐ligand 1 (PD‐1/PD‐L1) and cytotoxic T‐lymphocyte‐associated protein 4 (CTLA‐4) immune checkpoint pathways [9]. PD‐1/PD‐L1 pathway inhibitors such as pembrolizumab and nivolumab have been approved for second‐line or later treatment of PD‐L1‐positive or advanced ESCC patients, which demonstrate significant antitumor activity [8].

With the expanding clinical application of ICIs in treating ESCC, researchers are increasingly focusing on optimizing treatment strategies and identifying potential beneficiary populations. Over the past few years, the exploration of noninvasive prognostic biomarkers has become a crucial research direction [10, 11, 12, 13, 14]. Among them, the C‐PLAN index, as a newly developed prognostic prediction model, integrates multiple clinical indices, such as C‐reactive protein (CRP), performance status (PS) [7], lactate dehydrogenase (LDH), albumin (ALB), as well as the derived neutrophil‐to‐lymphocyte ratio (dNLR) [15]. While the C‐PLAN index has demonstrated robust prognostic value in predicting outcomes for non‐small cell lung cancer patients receiving immunotherapy [15, 16], its applicability in advanced ESCC populations requires further validation.

Currently, there exists limited published research investigating the C‐PLAN index specifically in advanced ESCC patients undergoing ICI therapy. This study therefore aimed to analyze the prognostic significance of the C‐PLAN index in advanced ESCC patients undergoing ICI therapy, with the goal of providing novel clinical references to guide treatment decision‐making for this patient population.

## 2 Materials and Methods

### 2.1 Study Population

A retrospective analysis was carried out on 292 advanced ESCC patients who received initial ICI therapy at Guangyuan Central Hospital between February 2020 and January 2023. After applying predefined inclusion and exclusion criteria, 241 patients were ultimately enrolled as study subjects. The C‐PLAN index was calculated based on LDH, CRP, PS, ALB, as well as dNLR. Patients were categorized into the Good group (C‐PLAN index: 0–1) and the Poor group (C‐PLAN index: 2–5). This study was approved by the ethical committee of Guangyuan Central Hospital and complied with the Declaration of Helsinki principles.

### 2.2 Inclusion and Exclusion Criteria

*Inclusion criteria*: (1) patients ≥ 18 years; (2) patients pathologically confirmed with ESCC; (3) patients staged as III or IV clinically; (4) patients who received PD‐1/PD‐L1 immunotherapy; and (5) patients with required clinical and follow‐up records.

*Exclusion criteria*: (1) patients with other pathological types of EC; (2) patients with a history of other primary malignancies; (3) patients with a chronic use of medications affecting ICI efficacy (e.g., the use of systemic corticosteroids (prednisone > 10 mg/day) for more than 14 days within 30 days before ICI treatment, and the use of broad‐spectrum antibiotics for more than 3 days within 30 days before ICI treatment or cumulative use for more than 7 days); (4) patients who received fewer than two cycles of ICI therapy; and (5) patients with concurrent hematologic disorders or autoimmune diseases.

### 2.3 Clinical Data Collection

Baseline clinical and laboratory data were collected before initial immunotherapy administration, including age, sex, body mass index (BMI), smoking history, alcohol consumption history, LDH, CRP, PS, ALB, dNLR, PD‐L1 tumor proportion score (TPS), combined positive score (CPS), clinical stage, tumor differentiation, tumor diameter, and treatment strategy. Meanwhile, based on hematological indicators, systemic immune inflammatory index (SII) and prognostic nutritional index (PNI) were calculated. SII = (peripheral venous neutrophil count × platelet count)/lymphocyte count. A higher score was indicative of worse immune and inflammatory status. PNI = albumin concentration (g/L) + peripheral blood lymphocyte count (×10^9^/L) × 5, with a higher score representing better nutritional status. Follow‐up records within 2 years after initial immunotherapy administration were collected. Each treatment cycle lasted 3 weeks, with patient follow‐up conducted every 6–8 weeks. Tumor prognosis evaluation indices included progression‐free survival (PFS) and overall survival (OS). PFS was defined as the duration from ICI therapy initiation to either disease progression detected by imaging (enhanced CT) or laboratory indices (such as CEA and CA199), or the follow‐up cutoff date, and OS as the period from the first ICI administration date to either death from any cause or the follow‐up cutoff date.

### 2.4 Efficacy Assessment

The study endpoints were determined as the date of last follow‐up, death, or disease progression. Treatment responses were evaluated by referring to Response Evaluation Criteria in Solid Tumors version 1.1 (RECIST 1.1) [17], including complete response (CR), partial response (PR), stable disease (SD) as well as progressive disease (PD). Response rates were calculated as follows: objective response rate (ORR): CR + PR; disease control rate (DCR): CR + PR + SD.

### 2.5 Definition of the C‐PLAN Index

As previously introduced [15], the C‐PLAN index was calculated by summing points assigned for the following five parameters: (1) the serum CRP level (≥ 1.0 mg/dL = 1 point; < 1.0 mg/dL = 0 points); (2) the PS score (0–1 = 0 points; 2–4 = 1 point); (3) the LDH level (< 223 U/L = 0 points; ≥ 223 U/L = 1 point); (4) the serum ALB (≥ 3.5 g/dL = 0 points; < 3.5 g/dL = 1 point); (5) the dNLR (≥ 3.0 = 1 point; < 3.0 = 0 points). The total C‐PLAN score spanned from 0 to 5 points, with scores of 0–1 classified as agood prognosis (Good group) and scores of 2–5 classified as a poor prognosis (Poor group), as detailed in Table S1.

### 2.6 Statistical Analyses

Sample size estimation was performed through G*Power 3.1.9.7 (Heinrich Heine University Düsseldorf, Germany) with the following parameters: two‐tailed test, *α* = 0.05, power (1 − *β*) = 0.95, and a medium anticipated effect size (*d* = 0.5). The final sample size met the requirements for all statistical tests. Data analysis and visualization were conducted using SPSS 27.0 (IBM Corp., Armonk, NY, USA) and GraphPad Prism 9.5 (GraphPad Software Inc., San Diego, CA, USA) statistical software. Normality of continuous variables was assessed using the Kolmogorov–Smirnov test. Normally distributed data were expressed as mean ± standard deviation and their between‐group comparisons were performed via the independent sample *t* test. Non‐normally distributed data were presented as median (range) and their between‐group comparisons were performed via the Mann–Whitney *U* test. Counting data were reported as counts (percentages), and their between‐group comparisons were carried out via the *χ* ^2^ test. Kaplan–Meier (K‐M) curves with log‐rank tests were employed for evaluating the impact of the C‐PLAN index on disease progression or death in advanced ESCC patients following ICI therapy. Cox proportional hazard regression models were established for the identification of independent risk factors for progression or death following ICI therapy. A two‐tailed test was adopted, with statistical significance defined as *p* < 0.05.

## 3 Results

### 3.1 Clinical Baseline Data

The patients' baseline characteristics are summarized in Table 1. According to the American Joint Committee on Cancer (AJCC) staging system (eighth edition), 83 patients (34.44%) were classified as Stage III and 158 patients (65.56%) as Stage IV. Regarding therapy regimens, 26 patients (10.79%) received immunotherapy monotherapy, including 18 treated with camrelizumab, 2 with tislelizumab, and 6 with toripalimab. The remaining 215 patients (89.21%) received combined treatment of immunotherapy with platinum/paclitaxel/radiotherapy, including 81 patients (33.61%) treated with pemetrexed‐platinum plus pembrolizumab, 4 (1.66%) with carboplatin‐pemetrexed plus atezolizumab, 3 (1.24%) with carboplatin‐paclitaxel plus atezolizumab, 12 (4.98%) with carboplatin‐paclitaxel‐bevacizumab plus atezolizumab, 67 (27.80%) with platinum‐paclitaxel plus pembrolizumab, 13 (5.39%) with carboplatin‐pemetrexed plus nivolumab–ipilimumab, 5 (2.07%) with carboplatin–paclitaxel plus nivolumab–ipilimumab, and 30 (12.45%) with intensity‐modulated radiotherapy plus camrelizumab. Of the patients, 70 patients (29.05%) had undergone prior surgical resection, while 171 (70.95%) had not received radical esophagectomy.

**TABLE 1:** Comparison of clinical data among patients.

Clinical baseline data	Patients in total (*n* = 241)
Age (years), median (range)	67 (51–81)
Sex (male/female, *n*)	165/76
BMI (kg/m^2^), median (range)	22.71 (17.65–27.94)
Smoking history (*n*, %)	191 (79.25)
Alcohol consumption history (*n*, %)	176 (73.03)
Laboratory results	
LDH (U/L), median (range)	227 (111–473)
CRP (mg/dL), median (range)	1.38 (0.09–27.88)
ALB (g/dL), median (range)	3.7 (1.6–4.7)
dNLR, median (range)	3.06 (0.70–9.70)
PS score (*n*, %)	
0–1 point	109 (45.23)
2–3 points	132 (54.77)
PD‐L1 TPS (*n*, %)	
≥ 10%	168 (69.71)
< 10%	73 (30.29)
CPS score (*n*, %)	
≥ 10%	195 (80.91)
< 10%	46 (19.09)
Clinical stage (*n*, %)	
III	83 (34.44)
IV	158 (65.56)
Differentiation (*n*, %)	
Low	138 (57.26)
Medium	96 (39.83)
High	7 (2.90)
Tumor diameter (cm), median (range)	3.7 (2.5–5.8)
Treatment strategy (*n*, %)	
Monotherapy	26 (10.79)
Combined therapy	215 (89.21)
Radiotherapy administered (cases, %)	
Yes	30 (12.45)
No	211 (87.55)
ICI treatment cycle (*n*, %)	
2–3	66 (27.39)
4–6	175 (72.61)
Surgical history (*n*, %)	
Yes	70 (29.05)
No	171 (70.95)

Abbreviations: ALB, albumin; BMI, body mass index; CRP, C‐reactive protein; dNLR, derived neutrophil‐to‐lymphocyte ratio; LDH, lactate dehydrogenase; PD‐L1, programmed death‐ligand 1; TPS, tumor proportion score.

### 3.2 Association of the C‐PLAN Index With Clinicopathological Characteristics of Advanced ESCC Patients

The C‐PLAN index was derived from five parameters: LDH, CRP, PS score, ALB, as well as dNLR. Patients were classified into two prognostic groups: the Good group (C‐PLAN score of 0–1) and the Poor group (C‐PLAN score of 2–5). An intergroup comparative analysis of clinicopathological characteristics demonstrated no notable differences in age, BMI, sex distribution, smoking history, alcohol consumption history, PD‐L1 TPS and CPS, tumor differentiation, treatment strategy, radiotherapy information, treatment cycle, and surgical history (all *p* > 0.05). However, notable inter‐group differences were found in clinical stage and tumor diameter between the two groups (both *p <* 0.05, Table 2).

**TABLE 2:** Comparison of clinical data between groups.

Clinical baseline data	Good group (*n* = 75)	Poor group (*n* = 166)	*p*
Age (years), median (range)	67 (51–80)	67 (54–81)	0.654
Sex (male/female, *n*)	53/22	112/54	0.621
BMI (kg/m^2^)	23.13 ± 2.22	22.59 ± 2.17	0.073
Smoking history (*n*, %)	56 (74.67)	135 (81.33)	0.238
Alcohol consumption history (*n*, %)	49 (65.33)	127 (76.51)	0.070
PD‐L1 TPS (*n*, %)			
≥ 10%	57 (76.00)	111 (66.87)	0.153
< 10%	18 (24.00)	55 (33.13)	
CPS score (*n*, %)			
≥ 10%	63 (84.00)	132 (79.52)	0.412
< 10%	12 (16.00)	34 (20.48)	
Clinical stage (*n*, %)			
III	33 (44.00)	50 (30.12)	0.036
IV	42 (56.00)	116 (69.88)	
Differentiation (*n*, %)			
Low	41 (54.67)	97 (58.43)	0.832
Medium	32 (42.67)	64 (38.55)	
High	2 (2.67)	5 (3.01)	
Tumor diameter (cm)	3.38 ± 0.41	3.97 ± 0.56	< 0.001
Treatment strategy (*n*, %)			
Monotherapy	9 (12.00)	17 (10.24)	0.684
Combined therapy	66 (88.00)	149 (89.76)	
*Treatment cycle (n, %)*			
Radiotherapy administered (cases, %)			
Yes	8 (10.67)	22 (13.25)	0.573
No	67 (89.33)	144 (86.75)	
2–3	16 (21.33)	50 (30.12)	0.157
4–6	59 (78.67)	116 (69.88)	
Surgical history (*n*, %)			
Yes	24 (32.00)	46 (27.71)	0.497
No	51 (68.00)	120 (72.29)	

*Note*: Normality of continuous variables was assessed using the Kolmogorov–Smirnov test. Normally distributed data were expressed as mean ± standard deviation and their between‐group comparisons were conducted using the independent sample *t* test. Non‐normally distributed data were presented as median (minimum, maximum) values and their between‐group comparisons were performed using the Mann–Whitney *U* test. Counting data were reported as counts (percentages), and their between‐group comparisons were conducted using the *χ* ^2^ test.

Abbreviations: BMI, body mass index; CPS, combined positive score; PD‐L1, programmed death‐ligand 1; TPS, tumor proportion score.

### 3.3 Association of the C‐PLAN Index With the Response of Advanced ESCC Patients to ICI Therapy

The treatment response to ICIs in advanced ESCC patients was assessed. The analysis demonstrated notably higher ORR and DCR in the Good group than in the Poor group (both *p* < 0.05, Table 3).

**TABLE 3:** Association of the C‐PLAN index with the response of advanced ESCC patients to ICI therapy.

Efficacy	Good group (*n* = 75) (*n*, %)	Poor group (*n* = 166) (*n*, %)	*p*
CR	3 (4.00)	2 (1.20)	—
PR	13 (17.33)	5 (3.01)	—
SD	30 (40.00)	7 (4.22)	—
PD	29 (38.67)	152 (91.57)	—
ORR	16 (21.33)	7 (4.22)	< 0.001
DCR	46 (61.33)	14 (8.43)	< 0.001

*Note*: Counting data were reported as counts (percentages), and their between‐group comparisons were conducted using the *χ* ^2^ test.

Abbreviations: CR, complete response; DCR, disease control rate; ORR, objective response rate; PD, progressive disease; PR, partial response; SD, stable disease.

### 3.4 Association of the C‐PLAN Index With the Prognosis of Advanced ESCC Patients After ICI Therapy

The prognosis of all patients within 2 years after receiving ICI therapy was analyzed, and the results demonstrated a notably lower progression rate and mortality rate in the Good group than in the Poor group (both *p* < 0.001, Table 4).

**TABLE 4:** Association of the C‐PLAN index with the prognosis of advanced ESCC patients receiving ICI therapy.

Prognosis		Good group (*n* = 75) (*n*, %)	Poor group (*n* = 166) (*n*, %)	*p*
Disease progression status	Disease progression	29 (38.67)	152 (91.57)	< 0.001
	No progression	46 (61.33)	14 (8.43)	
Survival status	Alive	53 (70.67)	43 (25.90)	< 0.01
	Deceased	22 (29.33)	123 (74.10)	

*Note*: Counting data were reported as counts (percentages), and their between‐group comparisons were conducted using the *χ* ^2^ test.

### 3.5 An Elevated C‐PLAN Index Increases the Risk of Disease Progression or Death in Advanced ESCC Patients After ICI Therapy

**FIGURE 1:**
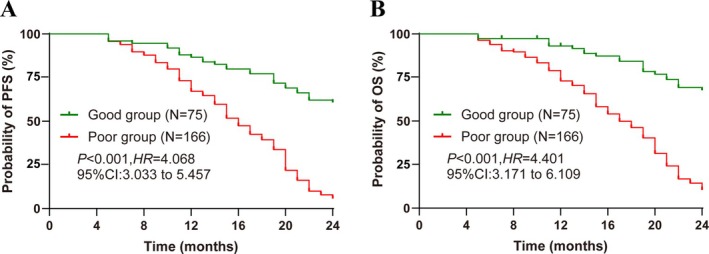
Survival analysis curves of advanced ESCC patients treated with ICIs. (A) PFS. (B) OS.

The prognostic value of the C‐PLAN index was further analyzed using K‐M curves. According to the results, the Good group had notably longer PFS and OS than the Poor group (both *p* < 0.001), indicating that a high C‐PLAN index (2–5 points) increased the risk of disease progression or death in advanced ESCC patients following ICI therapy (Figure 1).

### 3.6 An Elevated C‐PLAN Index Serves as an Independent Risk Factor for Disease Progression or Death in Advanced ESCC Patients Receiving ICI Therapy

To precisely evaluate the impacts of the C‐PLAN index on PFS and OS in advanced ESCC patients, Cox regression analyses were conducted using disease progression status (0 = no progression, 1 = progression) and survival status (0 = alive, 1 = deceased) as dependent variables. The following covariates were included as independent variables: age, sex, BMI, smoking history, alcohol consumption history, PD‐L1 TPS, CPS, clinical stage, tumor differentiation, tumor diameter, treatment strategy, radiotherapy information, treatment cycle, and surgical history. Variables with *p* < 0.05 in the univariate analysis were subsequently incorporated into the multivariate Cox regression model. The results identified PD‐L1 CPS < 10%, Clinical Stage IV, and high C‐PLAN index (2–5 points) as independent risk factors for disease progression or death in advanced ESCC patients following ICI therapy (all *p* < 0.05, Tables 5 and 6).

**TABLE 5:** Analysis of independent risk factors for disease progression in advanced ESCC patients receiving ICI therapy.

Univariate analysis				Multivariate analysis		
Items	*p*	HR	95% CI	*p*	HR	95% CI
Age (year)	0.730	0.995	0.968–1.023	—	—	—
Sex (female = 0, male = 1)	0.688	0.939	0.689–1.279	—	—	—
BMI (kg/m^2^)	0.712	0.987	0.923–1.056	—	—	—
Smoking history (no = 0, yes = 1)	0.090	1.398	0.949–2.058	—	—	—
Alcohol consumption history (no = 0, yes = 1)	0.493	1.125	0.803–1.577	—	—	—
PD‐L1 TPS (≥ 10% = 0, < 10% = 1)	0.007	1.537	1.127–2.096	0.325	1.500	0.647–3.711
PD‐L1 CPS (≥ 10% = 0, < 10% = 1)	0.019	3.221	1.210–8.578	0.019	4.174	1.270–13.722
Clinical stage (III = 0, IV = 1)	< 0.001	2.096	1.488–2.952	< 0.001	8.901	3.701–21.405
Differentiation (high vs. medium)	0.287	0.635	0.275–1.466	—	—	—
Differentiation (high vs. low)	0.344	0.671	0.294–1.532	—	—	—
Tumor diameter	< 0.001	1.815	1.416–2.326	0.742	1.153	0.494–2.694
Treatment strategy (monotherapy = 0, combined therapy = 1)	0.102	1.556	0.917–2.643	—	—	—
Radiotherapy (no = 0, yes = 1)	0.229	0.747	0.464–1.201	—	—	—
Treatment cycle (4–6 = 0, 2–3 = 1)	0.601	0.842	0.443–1.602	—	—	—
Surgical history (no = 0, yes = 1)	0.430	0.878	0.636–1.212	—	—	—
C‐PLAN index (0–1 = 0, 2–5 = 1)	< 0.001	4.425	2.945–6.649	< 0.001	20.506	7.425–56.630

**TABLE 6:** Analysis of independent risk factors for death in advanced ESCC patients receiving ICI therapy.

Univariate analysis				Multivariate analysis		
Item	*p*	HR	95% CI	*p*	HR	95% CI
Age (year)	0.496	0.989	0.960–1.020	—	—	—
Sex (female = 0, male = 1)	0.881	0.974	0.688–1.379	—	—	—
BMI (kg/m^2^)	0.250	0.957	0.887–1.032	—	—	—
Smoking history (no = 0, yes = 1)	0.757	1.065	0.716–1.584	—	—	—
Alcohol consumption history (no = 0, yes = 1)	0.526	1.130	0.775–1.648	—	—	—
PD‐L1 TPS (≥ 10% = 0, < 10% = 1)	0.021	1.505	1.063–2.131	0.472	1.277	0.656–2.489
D‐L1CPS (≥ 10% = 0, < 10% = 1)	0.037	2.144	1.047–4.390	0.017	2.745	1.201–6.274
Clinical stage (III = 0, IV = 1)	< 0.001	2.850	1.881–4.318	< 0.001	6.785	3.463–13.295
Differentiation (high vs. medium)	0.265	0.593	0.237–1.486	—	—	—
Differentiation (high vs. low)	0.341	0.644	0.260–1.592	—	—	—
Tumor diameter	< 0.001	1.857	1.407–2.450	0.820	1.073	0.585–1.968
Treatment strategy (monotherapy = 0, combined therapy = 1)	0.134	1.573	0.870–2.843	—	—	—
Radiotherapy (no = 0, yes = 1)	0.858	0.957	0.591–1.550	—	—	—
Treatment cycle (4–6 = 0, 2–3 = 1)	0.424	0.792	0.446–1.405	—	—	—
Surgical history (no = 0, yes = 1)	0.751	0.944	0.663–1.346	—	—	—
C‐PLAN index	< 0.001	4.828	3.032–7.689	< 0.001	7.203	3.352–15.479

### 3.7 The C‐PLAN Index Can Assist in Predicting Disease Progression or Death in Patients With Advanced ESCC Receiving ICI Therapy

**FIGURE 2:**
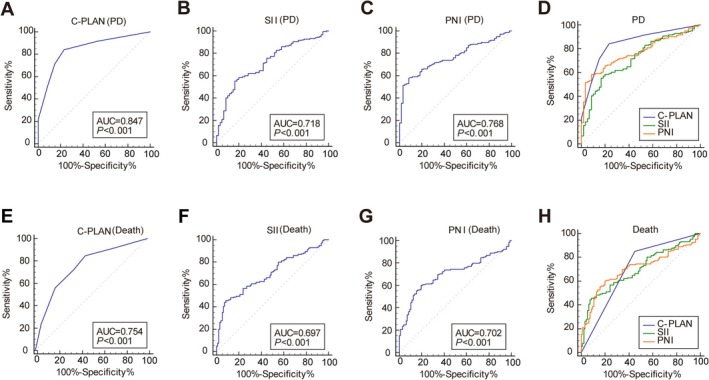
ROC curve analysis of various indicators to assist in predicting disease progression or death in patients with advanced ESCC. (A–D) ROC curves were plotted to analyze the predictive value of C‐PLAN index, SII, and PNI for disease progression in patients with advanced ESCC. (E–H) ROC curves to analyze the predictive value of C‐PLAN index, SII, and PNI for 2‐year death in patients with advanced ESCC after ICI treatment. The expression level corresponding to the maximum value of the Youden index was selected as the optimal threshold. PD, progressive disease.

The predictive value of the C‐PLAN index for disease progression or death in advanced ESCC patients was analyzed by plotting receiver operating characteristic (ROC) curves. The results demonstrated that the areas under the curve (AUCs) of the C‐PLAN index assisting prediction of disease progression and death in advanced ESCC patients within 2 years after ICI treatment were 0.847 (95% CI: 0.795–0.890, cutoff value of 1, sensitivity of 83.98%, specificity of 76.67%) and 0.754 (95% CI: 0.694–0.807, cutoff value of 1, sensitivity of 84.83%, specificity of 55.21%), respectively (Figure 2A–E). In addition, the C‐PLAN index was compared with traditional prognostic indicators (SII and PNI). The AUCs of SII assisting prediction of disease progression and death in patients with advanced ESCC within 2 years after ICI treatment were 0.718 (95% CI: 0.657–0.774, cutoff value: 681.44, sensitivity: 55.25%, specificity: 83.33%) and 0.697 (95% CI: 0.635–0.754, cutoff value: 705.85, sensitivity: 45.52%, specificity: 91.67%), respectively (Figure 2B–F). The AUCs of PNI assisting prediction of disease progression and death in patients with advanced ESCC within 2 years after ICI treatment were 0.768 (95% CI: 0.709–0.819, cutoff value of 43.77, sensitivity of 58.01%, specificity of 91.67%) and 0.702 (95% CI: 0.640–0.759, cutoff value of 43.03, sensitivity of 60.00%, specificity of 80.21%), respectively (Figure 2C–G). These results suggested that the C‐PLAN index and traditional prognostic indicators (SII and PNI) had high predictive power for disease progression or death in patients with advanced ESCC after ICI treatment. Further comparisons of AUCs among the three using MedCalc software showed that the C‐PLAN index had a higher AUC for predicting disease progression in patients with ESCC after ICI treatment than SII and PNI detection (*p* < 0.001, *p* = 0.028, Figure 2D). However, its AUC for predicting death within 2 years after ICI treatment in patients with ESCC was only slightly higher than SII and PNI tests, and the difference was not statistically significant (*p* > 0.05, Figure 2H).

## 4 Discussion

Prior research has suggested ICIs, particularly PD‐1/PD‐L1 inhibitors, as first‐line therapy for advanced EC, which greatly prolong PFS and OS of patients [18]. However, despite the revolutionary breakthrough ICIs have brought to advanced cancer treatment, clinical observations indicate their ORR remains limited to 15%–60% [19], meaning a substantial proportion of patients fail to derive significant clinical benefit. This heterogeneity in treatment response underscores the urgent need to identify reliable predictive biomarkers for optimal patient selection. Multiple studies [20, 21, 22] have confirmed that inflammatory indices including the platelet‐to‐lymphocyte ratio, neutrophil‐to‐lymphocyte ratio, systemic immune‐inflammation index, as well as PNI can predict immunotherapy efficacy in EC. For instance, lower PNI is strongly linked to worse PFS (HR = 0.35) and OS (HR = 0.41) [21]. Nevertheless, no single clinical parameter can accurately predict response to ICIs, highlighting the critical importance of developing multifactorial predictive models incorporating multiple clinical indices.

This study developed the C‐PLAN index based on LDH, CRP, PS score, ALB, as well as dNLR, all of which have been previously documented to correlate with ICI efficacy in EC. Li et al. [23] have demonstrated that the serum LDH level can serve as a powerful independent predictor for both PFS (*p* = 0.023) and OS (*p* < 0.001) in advanced ESCC patients undergoing anti‐PD‐1 therapy. Another study by Li et al. [24] has revealed that elevated pretreatment CRP/ALB is strongly bound up with worse OS in advanced ESCC patients who received immunotherapy. Da et al. [22] conducted a study on 162 advanced ESCC patients receiving anti‐PD‐1 therapy and revealed ECOG PS ≥ 2 as an independent poor prognostic factor affecting both OS and PFS with the multivariate regression analysis. Additionally, Kuang et al. [25] have identified baseline serum ALB level (*p* = 0.003) and dNLR (*p* = 0.003) as independent predictors of survival outcomes in immunotherapy for small cell lung cancer. In the current study, the Good and Poor groups had notable differences regarding clinical stage and tumor diameter. Notably, the Good group demonstrated superior clinical benefits: markedly higher ORR and DCR, markedly lower risks of disease progression and mortality, as well as markedly longer PFS and OS than the Poor group. The multivariate analysis further confirmed high C‐PLAN index (2–5 points) and clinical stage IV as independent risk factors for patient death. These results indicate that the C‐PLAN index, as a comprehensive scoring system integrating inflammatory status, nutritional condition, and tumor burden, provides more accurate prediction of immunotherapy efficacy compared to single‐parameter indice, offering reliable evidence to guide clinical decision‐making. Moreover, when compared with the traditional prognostic indicators (SII and PNI), the C‐PLAN index had a higher AUC for predicting disease progression in patients with ESCC after ICI treatment, and a slightly higher AUC for predicting death with no statistically significance. The establishment of this index not only facilitates the identification of potential beneficiaries from immunotherapy, but also provides critical guidance for developing personalized treatment strategies for high‐risk patients, demonstrating broad clinical application prospects.

Based on current evidence, the components of the C‐PLAN index likely influence immunotherapy outcomes through multiple interconnected mechanisms: First, CRP promotes systemic inflammation by inducing interleukin‐1 beta and tumor necrosis factor‐alpha release, while elevated neutrophils (reflected in dNLR) upregulate PD‐L1 expression to suppress T‐cell function. These effects synergistically disrupt immune microenvironment homeostasis [26, 27]. Second, as a key glycolytic enzyme, an elevated LDH leads to lactate accumulation, impairing the T/NK cell function. Additionally, poor PS correlates with increased serum PGE2 levels, which promotes immune evasion by regulating Th cell differentiation and NK cell activity [28, 29]. Additionally, hypoalbuminemia (reduced ALB) may compromise FcRn‐mediated IgG metabolism, thereby reducing PD‐1 inhibitor drug exposure [30, 31]. These mechanisms collectively establish an “inflammation‐metabolism‐pharmacokinetics” multidimensional regulatory network, wherein CRP/dNLR primarily drives pro‐inflammatory microenvironment formation, LDH/PGE2 mediates immunosuppressive metabolic reprogramming, and ALB modulates drug metabolic processes—ultimately converging to influence tumor immune evasion and ICI therapeutic efficacy through multiple pathways. These findings provide a comprehensive biological foundation for understanding the prognostic significance of the C‐PLAN index, and also illuminate potential directions for developing targeted combination therapeutic strategies.

As a single‐center retrospective analysis, this study has preliminarily demonstrated the prognostic significance of the C‐PLAN index for immunotherapy efficacy in advanced ESCC, but the following limitations should be acknowledged. First, the single‐center retrospective design with a limited sample size carries inherent potential biases. Additionally, the relatively short follow‐up duration necessitates extended observation periods to further elucidate the impact of the C‐PLAN index on long‐term outcomes in advanced ESCC patients following ICI therapy. To enhance the robustness of our findings, we propose a three‐pronged approach: (1) expanding the sample size, (2) including different groups (such as patients from different countries, races, and undergoing different treatment strategies to make the relevant research results more widely applicable), (3) initiating multicenter prospective studies, and (4) extending follow‐up duration to further improve the external validity and clinical applicability of the research results. In addition, in this study, we focused on advanced ESCC patients receiving ICI treatment, aiming to further explore the prognostic value of the C‐PLAN index in this specific cancer type and specific treatment context. We plan to gradually expand our research scope in future studies to include other common cancer types such as lung cancer, gastric cancer, and so forth, in order to systematically evaluate the prognostic efficacy and clinical practicality of the C‐PLAN index in different cancers. These measures will significantly improve the statistical power and reliability of our conclusions.

In summary, the C‐PLAN index demonstrates strong associations with clinical stage and tumor diameter in advanced ESCC patients, and an elevated C‐PLAN index (2–5 points) increases the risk of disease progression or death following ICI therapy. This discovery helps clinical doctors to more accurately identify high‐risk patients when formulating treatment plans, and thus adopt more personalized treatment strategies. For example, for patients with a high C‐PLAN index, closer follow‐up monitoring or combination with other treatment methods may be needed to improve patients' prognosis. As a robust prognostic biomarker, this C‐PLAN index provides an objective basis for survival assessment and treatment strategy optimization in patients with advanced ESCC.

## Ethics Statement

This study was approved by the ethical committee of Guangyuan Central Hospital and complied with the Declaration of Helsinki principles.

## Consent

All patients were informed of the purpose of the study and signed an informed consent form (approval number: GYZXLLH2025010).

## Conflicts of Interest

The authors declare no conflicts of interest.

## Supporting information


**Table S1:** The C‐PLAN index.

## Data Availability

The data that support the findings of this study are available from the corresponding author upon reasonable request.
