# Supplementary material for: Prognostic Value of the C‐PLAN Index in Advanced Esophageal Squamous Cell Carcinoma Patients Treated With Immune Checkpoint Inhibitors
Source: Kaohsiung J Med Sci. 2025 Dec 4;42(6):e70140. doi: 10.1002/kjm2.70140 (PMC13248814; doi:10.1002/kjm2.70140)
Supplement: Supplementary file 1 — Table S1: The C‐PLAN index. [file KJM2-42-e70140-s001.doc]

**Supplementary Table 1** The C-PLAN index

| Category | Score 0 | Score 1 |
| --- | --- | --- |
| CRP (mg/dL) | < 1.0 | ≥ 1.0 |
| PS | 0-1 | 2-4 |
| LDH (U/L) | < 223 | ≥ 223 |
| ALB (g/dL) | ≥ 3.5 | < 3.5 |
| dNLR | < 3.0 | ≥ 3.0 |
